# *BRPF1*‐associated intellectual disability, ptosis, and facial dysmorphism in a multiplex family

**DOI:** 10.1002/mgg3.665

**Published:** 2019-04-24

**Authors:** Naomi Pode‐Shakked, Ortal Barel, Ben Pode‐Shakked, Aviva Eliyahu, Amihood Singer, Omri Nayshool, Nitzan Kol, Annick Raas‐Rothschild, Elon Pras, Mordechai Shohat

**Affiliations:** ^1^ Department of Pediatrics A Edmond and Lily Safra Children's Hospital, Sheba Medical Center Tel‐Hashomer Israel; ^2^ The Dr. Pinchas Borenstein Talpiot Medical Leadership Program Sheba Medical Center Tel‐Hashomer Israel; ^3^ Sackler Faculty of Medicine Tel‐Aviv University Tel‐Aviv Israel; ^4^ The Genomic Unit, Sheba Cancer Research Center Sheba Medical Center Tel‐Hashomer Israel; ^5^ The Danek Gertner Institute of Human Genetics Sheba Medical Center Tel‐Hashomer Israel; ^6^ Community Genetics Public Health Services, Ministry of Health Jerusalem Israel

**Keywords:** blepharophimosis, BRPF1, intellectual disability, ptosis

## Abstract

**Background:**

Over 500 epigenetic regulators have been identified throughout the human genome. Of these, approximately 30 chromatin modifiers have been implicated thus far in human disease. Recently, variants in *BRPF1*, encoding a chromatin reader, have been associated with a previously unrecognized autosomal dominant syndrome manifesting with intellectual disability (ID), hypotonia, dysmorphic facial features, ptosis, and/or blepharophimosis in 22 individuals.

**Patients and Methods:**

We report a multiply affected nonconsanguineous family of mixed Jewish descent who presented due to ID in three male siblings. Molecular analysis of the family was pursued using whole exome sequencing (WES) and subsequent Sanger sequencing.

**Results:**

Whole exome sequencing analysis brought to the identification of a novel heterozygous truncating mutation (c.556C>T, p.Q186*) in the *BRPF1 *gene in the affected siblings and their mother. The four affected individuals showed varying degrees of intellectual disability, distinct facial features including downslanted palpebral fissures, ptosis, and/or blepharophimosis. Their clinical characteristics are discussed in the context of previously reported patients with the *BRPF1*‐related phenotype.

**Conclusion:**

The reported family contributes to the current knowledge regarding this unique and newly recognized genetic disorder, and further implicates the role of *BRPF1* in human brain development.

## 1 INTRODUCTION

The growing use of next generation sequencing techniques has revolutionized the diagnostic odyssey for many families with intellectual disability, and continues to elucidate and implicate pathways and genes not previously associated with syndromic or nonsyndromic intellectual disability in humans.

A unique group of genes which have been associated with various phenotypes of intellectual disability is that of epigenetic regulators. Of these, several dozen chromatin regulators have been implicated, including lysine acetyltransferases, histone deacetylases, DNA methyltransferases, ATP‐dependent helicases and others. Several well‐recognized examples include *CREBBP* and *EP300*, both associated with Rubinstein‐Taybi syndrome (Grozeva et al., 2014); *KANSL1* associated with the 17q21.31 microdeletion (Koolen‐de Vries) syndrome (Kaiser et al., 2014); and *HDAC8* implicated in a Cornelia de Lange‐like phenotype (Koolen et al., 2012), among others. However, less is known regarding the association of chromatin readers with human disease (Kuechler et al., 2015).

One such chromatin reader now linked to a recognizable intellectual disability phenotype, is Bromodomain and PHD finger‐containing protein 1 (BRPF1; OMIM *602410). By its interaction with several histone acetyltransferases (KAT6A, KAT6B and HBO1), BRPF1 promotes histone acetylation (Laue et al., 2008; Li & Durbin, 2009). Most recently, two groups had simultaneously described and characterized a new autosomal dominant disorder manifesting with intellectual disability, ptosis, and/or blepharophimosis and additional features, in 22 individuals, caused by deleterious mutations in *BRPF1* (Li, Gui, & Kwan, 2012; Mattioli et al., 2017).

We describe herein four additional affected individuals of a single family, exhibiting intellectual disability of variable severity and distinct facial features, found to harbor a novel mutation in *BRPF1.*

## 2 MATERIALS AND METHODS

### 2.1 Patients

Patients were evaluated at the Sheba Medical Center, Tel‐Hashomer, Israel. Written informed consent was obtained from the affected individuals or their legal guardians for both genetic analysis and publication of patients' facial photographs. Approval for human subject research was obtained from the Institutional Review Board of the Sheba Medical Center.

### 2.2 Whole exome sequencing

Whole exome sequencing (WES) was performed using an Agilent v5 SureSelect capture kit and Illumina 2,500 sequencing technology. For each sample, paired end reads (2 × 100 bp) were obtained, processed, and mapped to the genome. We used the BWA‐MEM algorithm (version 0.7.12) (McKenna et al., 2010) for alignment of the sequence reads to the human reference genome (hg19). The HaplotypeCaller algorithm of GATK version 3.4 was applied for variant calling, as recommended in the best practice pipeline (Musselman, Lalonde, Côté, & Kutateladze, 2012). KGG‐seq v.08 was used for annotation of identified variants (Narahara et al., 1990) and in house scripts were applied for filtering based on family pedigree and local dataset of variants detected in previous sequencing projects.

## 3 RESULTS

### 3.1 Clinical characteristics

**Figure 1:**
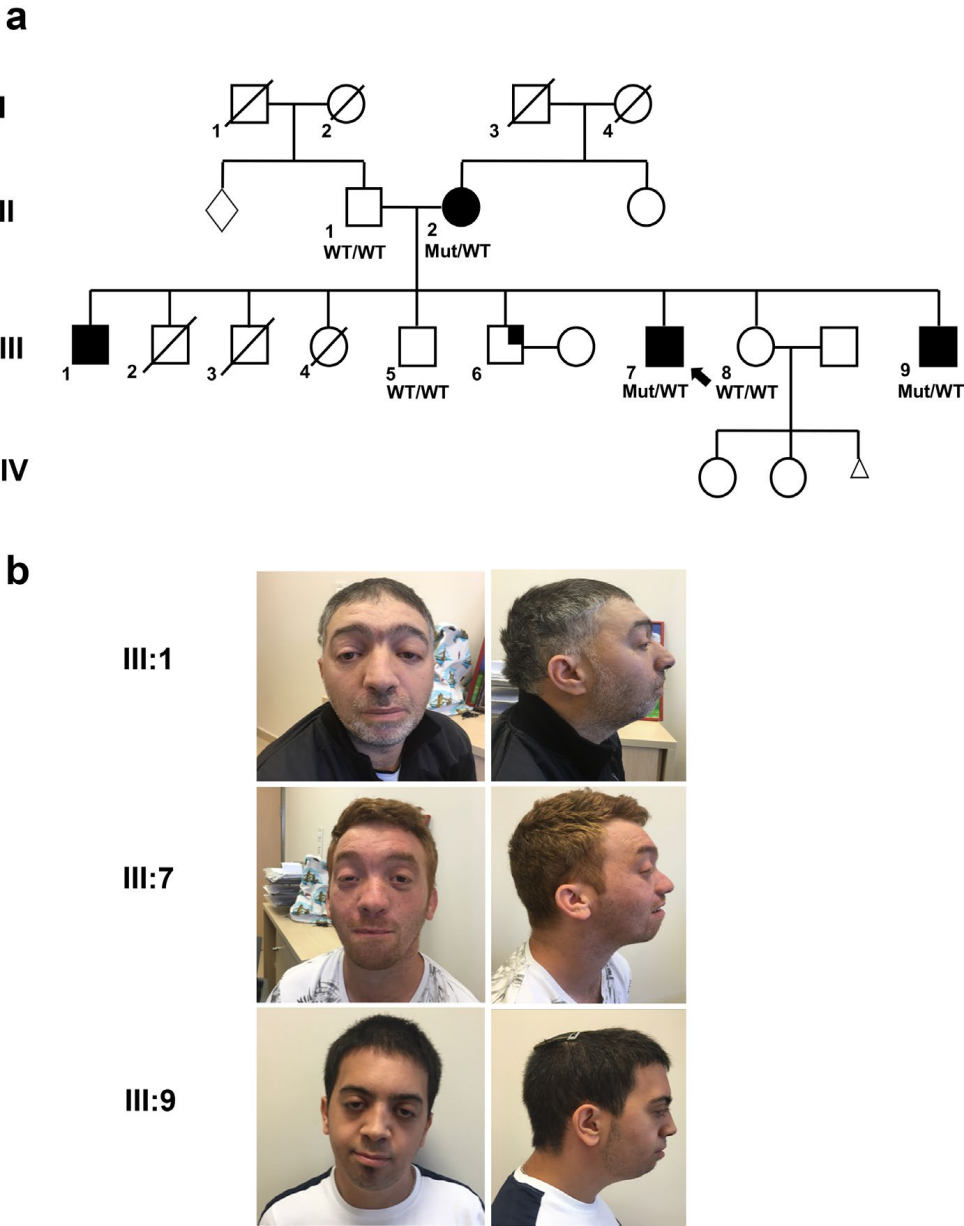
(a) Genogram of a multiply affected family with *BRPF1*‐associated phenotype. Proband is denoted by the arrow. Full symbols designate affected individuals. Partially full symbols designate individual with Leber's Hereditary Optic Neuropathy. Mut, allele harboring the p.Q186* mutation in *BRPF1. *WT, wild type allele. (b) Facial features of individuals harboring the heterozygous *BRPF1* variant. Note the ptosis, blepharophimosis, downslanted palpebral fissures and mild retrognathia

A nonconsanguineous family of mixed Jewish descent with three siblings affected with intellectual disability was referred to our Genetics Institute for evaluation, during an ongoing pregnancy of their healthy 27‐year‐old sister (Figure 1).

The proband (designated patient III:7) is a 30‐year‐old male, reported to have developmental delay and intellectual disability. As a child he required special schooling and currently finds employment in manual labor. He has never experienced seizures, nor visual or hearing deficits. Upon physical examination, he showed dysmorphic facial features, most notably bilateral ptosis, hypertelorism and downslanted palpebral fissures. Brain imaging was not performed, and previous genetic workup had included molecular studies for Fragile X (30 repeats) and a chromosomal microarray analysis considered to be normal.

Interestingly, two additional male siblings, 42 years old (patient III:1) and 25 years old (patient III:9) also share dysmorphic facial features including bilateral ptosis, downslanted palpebral fissures and retrognathia, and both have intellectual disability, with the eldest sibling (III:1) most severely affected. At 42 years of age, he is unemployed and dependent on his parents for daily living. The clinical characteristics of the affected individuals in the family are summarized in Table 1.

**Table 1:** Demographic and clinical characteristics in all individuals with *BRPF1* reported in the literature to date

Patient(s)	II:2	III:1	III:7	III:9	Mattioli et al. (*n* = 12)	Yan et al. (*n* = 10)	Total
Gender	F	M	M	F	6 M, 6 F		
Age at diagnosis (years)	61	42	30	24	3–37		
CNS Manifestations							
Intellectual disability	+	+	+	+	12/12	10/10	26/26
Neonatal feeding difficulties	NA	NA	NA	−	NA	4/9	4/10
Seizures	−	−	−	−	2/10	4−6/10	6−8/24
Neonatal hypotonia	NA	NA	NA	+	4/7	7/8	12/16
Brain MRI findings	NA	NA	NA	NA	2/4	3/7	5/11
Dysmorphic features							
Downslanting palpebral fissures	+	+	+	+	NA	4/10	8/14
Ptosis and/or blepharophimosis	+	+	+	+	11/11	6/10	21/25
Flat facial profile	−	−	−	−	NA	7/9	7/13

The family history is also notable for one male sibling (III:6) who had been previously diagnosed with Leber Hereditary Optic Neuropathy due to a m.3460G > A mutation in MT‐ND1, however is otherwise healthy and does not share his brothers' facial features nor intellectual impairment.

### 3.2 Molecular analysis

In order to reach a molecular diagnosis in the family, DNA was extracted from whole blood samples from the proband, his siblings and parents. WES was performed for the proband and his parents (trio WES), and had led to the identification of the previously unreported c.556C>T (p.Q186*) truncating mutation on exon 2 of the *BRPF1* gene (NM_004634), in the proband and his mother.

Further analysis of the mutation status in family members available for testing, revealed that the mutation fully segregated with the disease in the family (Figure 1), with affected individuals found to be heterozygous, and unaffected found wild type to the mutation.

## 4 DISCUSSION

The family described herein exhibited a variable phenotype, including intellectual disability of varying severity, accompanied by facial dysmorphic features and especially ptosis, blepharophimosis and dowslanting palpebral fissures. Due to limited medical and developmental history, and nonspecific findings upon physical examination, reaching an accurate clinical diagnosis or pursuing gene‐specific sequencing would have been extremely challenging. Through WES, however, a timely molecular diagnosis was reached, implicating a heterozygous variant in *BRPF1 *as the causative mutation in the family.

Consistent with the recent description of the *BRPF1*‐associated phenotype, the affected individuals showed developmental delay and mild to moderate intellectual disability (Li et al., 2012; Mattioli et al., 2017). Interestingly, at 61 years of age, their mother—reportedly healthy upon initial medical history—was also found to harbor the heterozygous pathogenic variant in *BRPF1*. Indeed, the mother (patient II:2) shares her sons' bilateral ptosis, and her cognitive status was self‐perceived as normal. This underscores both the variable neurodevelopmental phenotype of *BRPF1* haploinsuffficiency, as well as the importance of thorough history taking and accurate phenotyping of probands' family members, as her initial misclassification as unaffected (or partially affected) might have caused a misdiagnosis or misinterpretation of the WES findings.

Of note, terminal 3p deletions, as well as deletions of the 3p25‐p26 region, have been previously associated with a distinct microdeletion syndrome (OMIM #613792), manifesting with intellectual disability of variable severity, microcephaly, ptosis, and additional dysmorphic features (Park et al., 2014). Several genes are encompassed within these deletions, including *BRPF1 *and *SETD5*, with the majority of the phenotype previously considered to be attributed to the latter (Yan et al., 2017; You et al., 2015). Upon describing the *BRPF1*‐associated disorder, Mattioli and colleagues compared the phenotypes of individuals harboring point mutations or small deletions of *BRPF1* alone or SETD5 alone, to those with a 3p25 deletion encompassing both genes (Mattioli et al., 2017). Their data demonstrated that microcephaly and ptosis (either unilateral or bilateral) and/or blepharophimosis were significantly more common in those with *BRPF1* disruptions, while strabismus and small stature were enriched in this group, however did not reach statistical significance. The features seen in the affected individuals in the family described herein, harboring a pathogenic *BRPF1* variant, are consistent with these findings, further validating the previous conclusion that several specific phenotypic characteristics, including ptosis and blepharophimosis, are mainly attributable to *BRPF1* haploinsufficiency.

To conclude, *BRPF1* haploinsufficiency is an underdiagnosed cause of intellectual disability of variable severity, ptosis and/or blepharophimosis and additional nonspecific features, and should be considered in relevant clinical circumstances.

## CONFLICT OF INTEREST

All authors declare no conflict of interests.
